# The shared mechanism and potential diagnostic markers for premature ovarian failure and dry eye disease

**DOI:** 10.1038/s41598-024-67284-3

**Published:** 2024-07-13

**Authors:** Xi Long, Zixuan Wu, Pengfei Jiang, Kang Tan, Pei Liu, Qinghua Peng

**Affiliations:** 1 Hunan University of Chinese Medicine, Changsha, China; 2 The First Affiliated Hospital of Hunan University of Chinese Medicine, Changsha, China; 3 Quzhou Hospital of Zhejiang Medical and Health Group, Quzhou, China

**Keywords:** Premature ovarian failure, Dry eye disease, Shared mechanism, Potential diagnostic markers, Bioinformatics, Biomarkers, Medical research

## Abstract

Premature ovarian failure (POF), which is often comorbid with dry eye disease (DED) is a key issue affecting female health. Here, we explored the mechanism underlying comorbid POF and DED to further elucidate disease mechanisms and improve treatment. Datasets related to POF (GSE39501) and DED (GSE44101) were identified from the Gene Expression Omnibus (GEO) database and subjected to weighted gene coexpression network (WGCNA) and differentially expressed genes (DEGs) analyses, respectively, with the intersection used to obtain 158 genes comorbid in POF and DED. Kyoto Encyclopedia of Genes and Genomes (KEGG) and Gene Ontology (GO) analyses of comorbid genes revealed that identified genes were primarily related to DNA replication and Cell cycle, respectively. Protein–Protein interaction (PPI) network analysis of comorbid genes obtained the 15 hub genes: CDC20, BIRC5, PLK1, TOP2A, MCM5, MCM6, MCM7, MCM2, CENPA, FOXM1, GINS1, TIPIN, MAD2L1, and CDCA3. To validate the analysis results, additional POF- and DED-related datasets (GSE48873 and GSE171043, respectively) were selected. miRNAs-lncRNAs-genes network and machine learning methods were used to further analysis comorbid genes. The DGIdb database identified valdecoxib, amorfrutin A, and kaempferitrin as potential drugs. Herein, the comorbid genes of POF and DED were identified from a bioinformatics perspective, providing a new strategy to explore the comorbidity mechanism, opening up a new direction for the diagnosis and treatment of comorbid POF and DED.

## Introduction

Premature ovarian failure (POF), formerly known as premature ovarian insufficiency (POI) or premature menopause, is characterized by the cessation of ovarian function before 40 years of age^1^. This condition is marked by hypoestrogenism and diminished follicular reserves, leading to menstrual irregularities, infertility, and diminished health-related quality of life^2^. POF is a diverse disease frequently overlooked during diagnosis, with a multifaceted etiology that includes genetic, iatrogenic, infectious, and autoimmune origins. Clinically, POF symptoms predominantly stem from estrogen deficiency and present as amenorrhea, oligomenorrhea, vasomotor symptoms (such as hot flashes and night sweats), sleep disturbances, vulvovaginal atrophy, urinary frequency changes, recurrent infections, and mood disturbances, including irritability and emotional instability^3^. Women with POF have increased risks of cardiovascular disease, dementia, cognitive decline, Parkinsonism, dry eye syndrome, and osteoporosis^4,5^. The psychological impact of POF is profound, necessitating counseling to address infertility^6^, self-esteem issues, and increased anxiety and depression.

Dry eye disease (DED), also known as keratoconjunctivitis sicca (KCS), dry eye syndrome (DES), ocular surface disease (OSD), or dysfunctional tear syndrome (DTS), is a common ophthalmic disorder. This syndrome, particularly common in individuals over the age of 48 years, arises from an imbalance in the preocular tear film, affecting roughly one in seven individuals in this age group. DED originates from dysfunction within the nasolacrimal unit (encompassing the nasolacrimal glands, corneal surface, and eyelids), which leads to compromised or inadequate tear film production^7^. A physiologically intact tear film is crucial for clear vision because it collaborates with the cornea to focus light on the retina. Additionally, it plays a vital role in lubricating the eye, clearing ocular debris, and sustaining nutrition and oxygenation of ocular structures^8^. Patients with DED often experience symptoms such as ocular burning, blurred vision, or pain, which substantially diminish their quality of life. Activities that require visual focus, such as reading or computer work, can be challenging^9^. Despite the availability of treatments to alleviate the symptoms of DED, they often fail to address the underlying causes. A diverse array of factors, including autoimmunity, hormonal imbalances, and adverse environmental conditions, have been implicated in the development of DED^10^. Notably, dry eye symptoms may occasionally signal undiagnosed systemic diseases, and timely interventions could avert potentially life-threatening complications.

**Figure 1:**
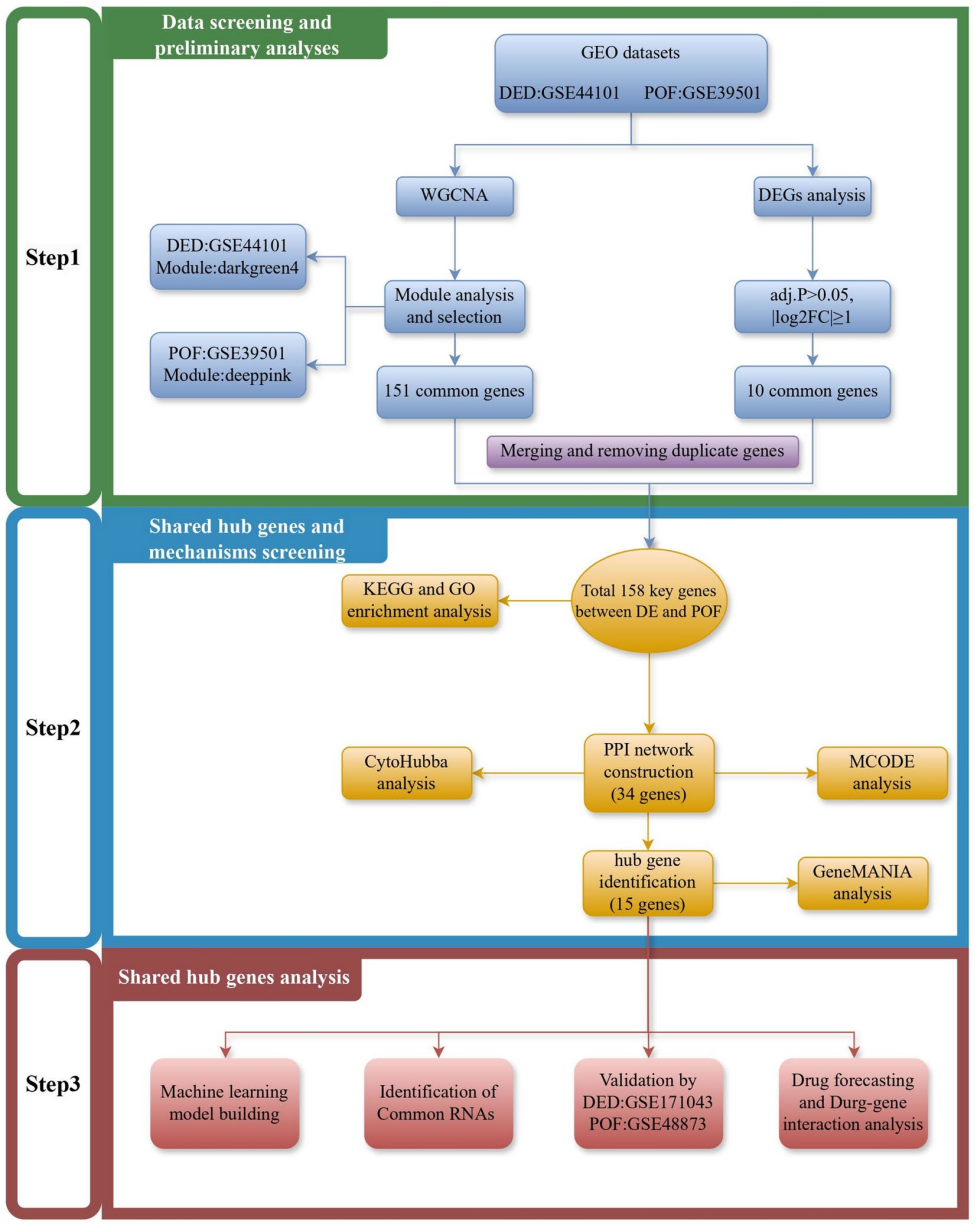
Schematic diagram of the general flow of this research work. In order to gain a more penetrating understanding of the mechanisms of comorbidity between POF and DED, we began our research journey using data from the GEO database. Specifically, this study was divided into three main parts. Firstly, in the first part we identified the GSE44101 and GSE39501 datasets as the primary cohorts through a careful matching strategy, and identified the GSE171043 and GSE48873 datasets for validation. We performed WGCNA analysis on GSE44101 and GSE39501 to identify the key modules and module genes, followed by differential expression analysis, respectively. The 158 common genes of POF and DED were obtained by merging and removing duplicate genes. Subsequently, in the second part, in order to reveal the functional basis of these common key genes, we performed exhaustive GO and KEGG analyses. Meanwhile, in order to further screen the hub genes, we executed a set of step-by-step screening of 15 hub genes after covering PPI, MCODE, Cytoscape, and GeneMANIA analyses. In the third part, the hub genes were further analysed and validated through seamless integration with 4 different methods and 2 datasets. This comprehensive approach not only enriches our understanding of the mechanisms of POF and DED comorbidity, but also lays a solid foundation for identifying prospective targeted therapies and interventions for the treatment of POF and DED comorbidity.

POF is frequently comorbid with DED, which markedly affects the physical and mental health of the affected women. Numerous studies have established a linkage between POF and DED^11,12^. The diminished estrogen levels resulting from ovarian hypoplasia adversely affect lacrimal gland secretion, leading to the characteristic symptoms of DED^13^. Additionally, certain inflammatory factors are aberrantly expressed under both conditions, suggesting a shared pathophysiological basis. Investigating these comorbid mechanisms is crucial for understanding the development and progression of both POF and DED^14,15^. Moreover, such explorations could offer novel perspectives and approaches for treating these conditions. The POF and DED Initiative, which utilizes high-throughput transcriptome sequencing and enrichment with detailed clinical annotations, presents an unparalleled opportunity to examine the transcriptional landscape and molecular pathways of POF and DED. Bioinformatics analyses of these comprehensive datasets have yielded valuable insights into their intricate pathophysiological mechanisms. However, a notable gap remains regarding the use of bioinformatics to explore the roles of differentially expressed genes (DEGs) in POF and DED. In the current study, we aimed to fill this gap by analyzing the POF and DED-related Gene Expression Omnibus (GEO) datasets to enhance our molecular understanding of POF and DED and identify new therapeutic targets, as illustrated in Fig. 1.

## Results

### GEO information

The DED- and POF-related datasets were screened from GEO to obtain gene chip datasets GSE44101 (microarray platform GPL1261), GSE39501 (microarray platform GPL6887), GSE171043 (microarray platform GPL28441), and GSE48873 (microarray platform GPL13607), which were eligible for the study. These datasets contain both normal and diseased samples. After screening, we selected the DED-related dataset GSE44101 for analysis and GSE171043 for validation, the POF-related dataset GSE39501 for analysis, and GSE48873 for validation.

### Weighted gene coexpression network analysis (WGCNA) of DED and POF

**Figure 2:**
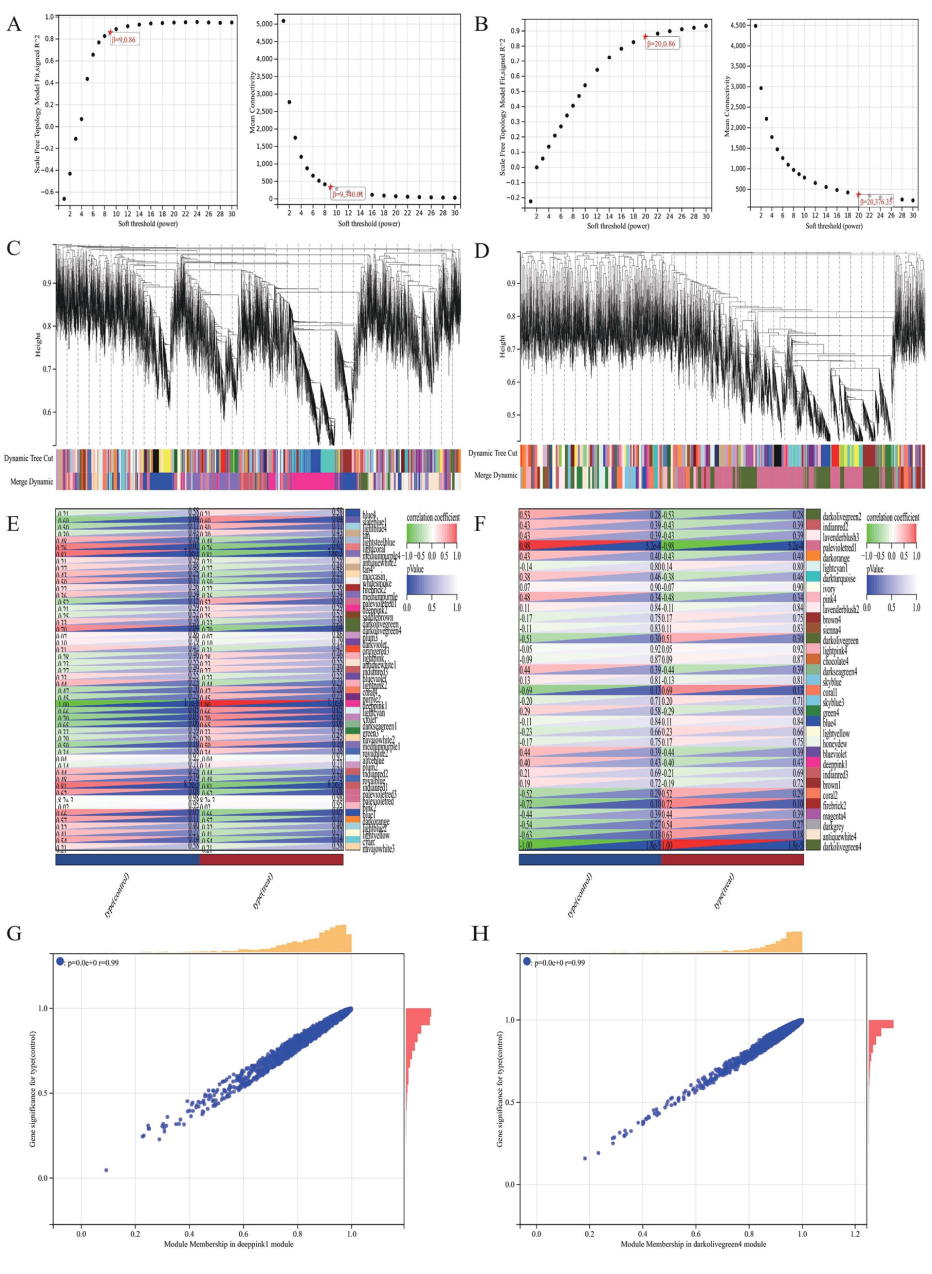
Weighted Gene Co-expression Network Analysis. (**A**) Selection of soft thresholds (β) for WGCNA analysis of POF. (**B**) Selection of soft thresholds (β) for WGCNA analysis of DED. (**C**) This diagram shows the degree of correlation within the module of the POF dataset. (**D**) This diagram shows the degree of correlation within the module of the DED dataset. (**E**) Relationship between modules and clinical features in POF dataset. (**F**) Relationship between modules and clinical features in DED dataset. Rows represent different modules and columns represent clinical features. Each cell contains the correlation between modules and clinical features and the corresponding *p*-value. (**G**) Module membership in deeppink1 module in POF dataset. (**H**) Module membership in darkolivegrenn4 module in DED dataset.

Data from the GSE44101 and GSE39501 datasets were analyzed after preprocessing to obtain sample clusters. The β values of 20 and 9 were selected as the soft threshold to construct a coexpression network respectively (Fig. 2A, B), which fulfilled the following conditions: (i) the scale-free fitting index was ˃ 0.8, (ii) the average connectivity of the network was good, and (iii) the network conformed to the non-scale topological network distribution. Module identification was performed using a dynamic tree-chopping algorithm and by merging similar modules. The gene sets within the modules were highly related. The final GSE44101 dataset obtained 33 modules, while the GSE39501 dataset obtained 50 modules, respectively (Fig. 2C, D). Upon correlating the modules of the GSE44101 and GSE39501 datasets with the clinical characteristics (treat and control) of the samples and setting the MM threshold to 0.8, GS threshold to 0.1, and weight threshold to 0.1, we found that the GSE44101 dataset darkolivegreen4 module was highly correlated with the clinical characteristics (r = 0.99, *P* = 0.00) of the tissues. The GSE39501 dataset deeppink1 module was highly correlated with clinical characteristics (r = 0.99, *P* = 0.00), as shown in Fig. 2E, F, G, H. Therefore, we selected the green GSE44101 dataset darkolivegreen4 module with the GSE39501 dataset deeppink1 module for further screening of key genes analysis. By analyzing WGCNA, 151 coexpressed genes in DED and POF were obtained by considering the intersection of the two (Fig. 4A).

### Analysis of DED and POF DEGs

**Figure 3:**
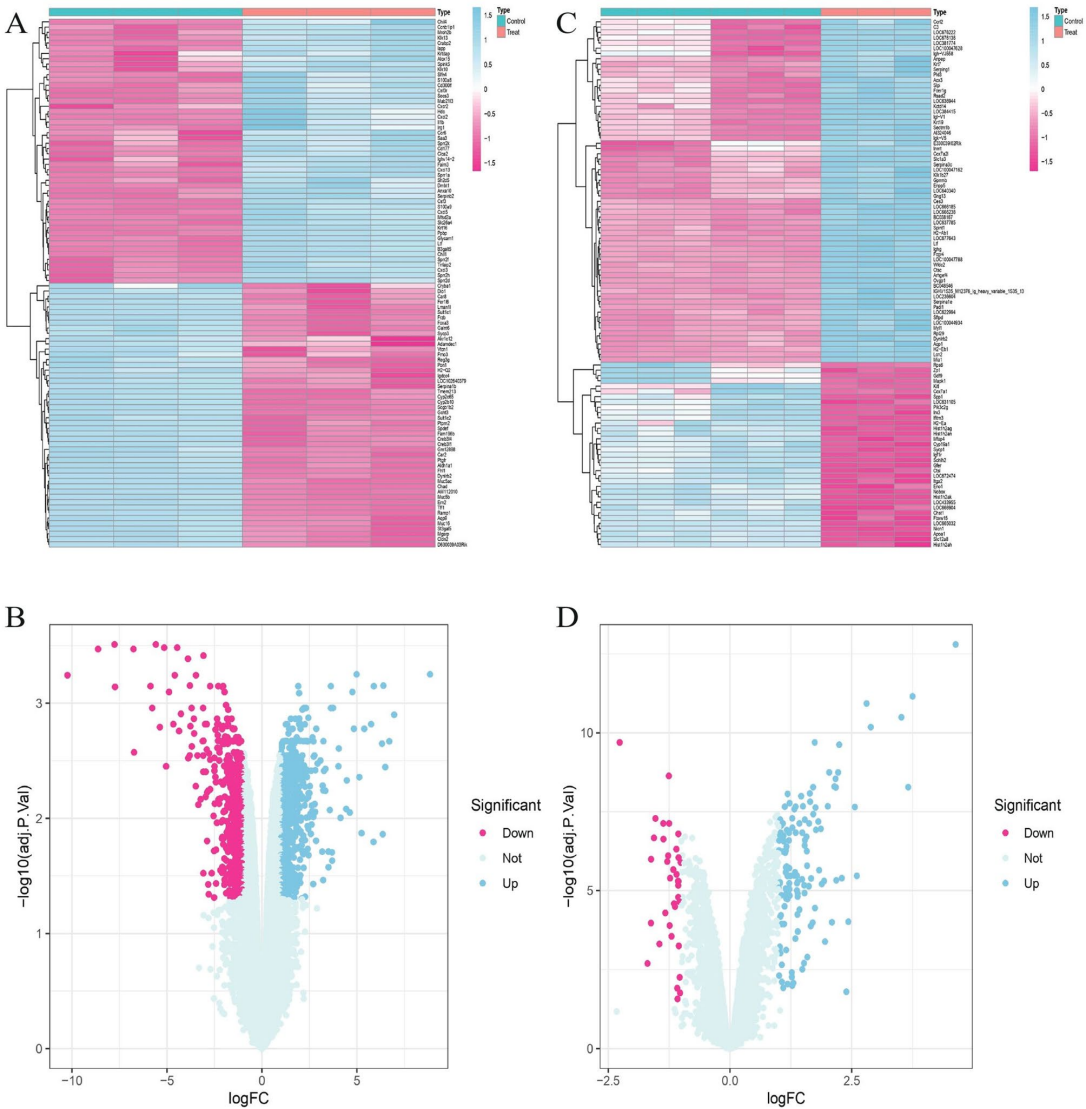
Differentially expressed genes in POF and DED. (**A**) Volcano plot of differentially expressed genes in DED dataset, with pink representing up-regulated genes and blue representing down-regulated genes. (**B**) Heatmap of differentially expressed genes in DED dataset. Pink color represents high expression and blue color represents low expression. (**C**) Volcano plot of differentially expressed genes in POF dataset, with pink representing up-regulated genes and blue representing down-regulated genes. (**D**) Heatmap of differentially expressed genes in POF dataset. Pink color represents high expression and blue color represents low expression.

**Figure 4:**
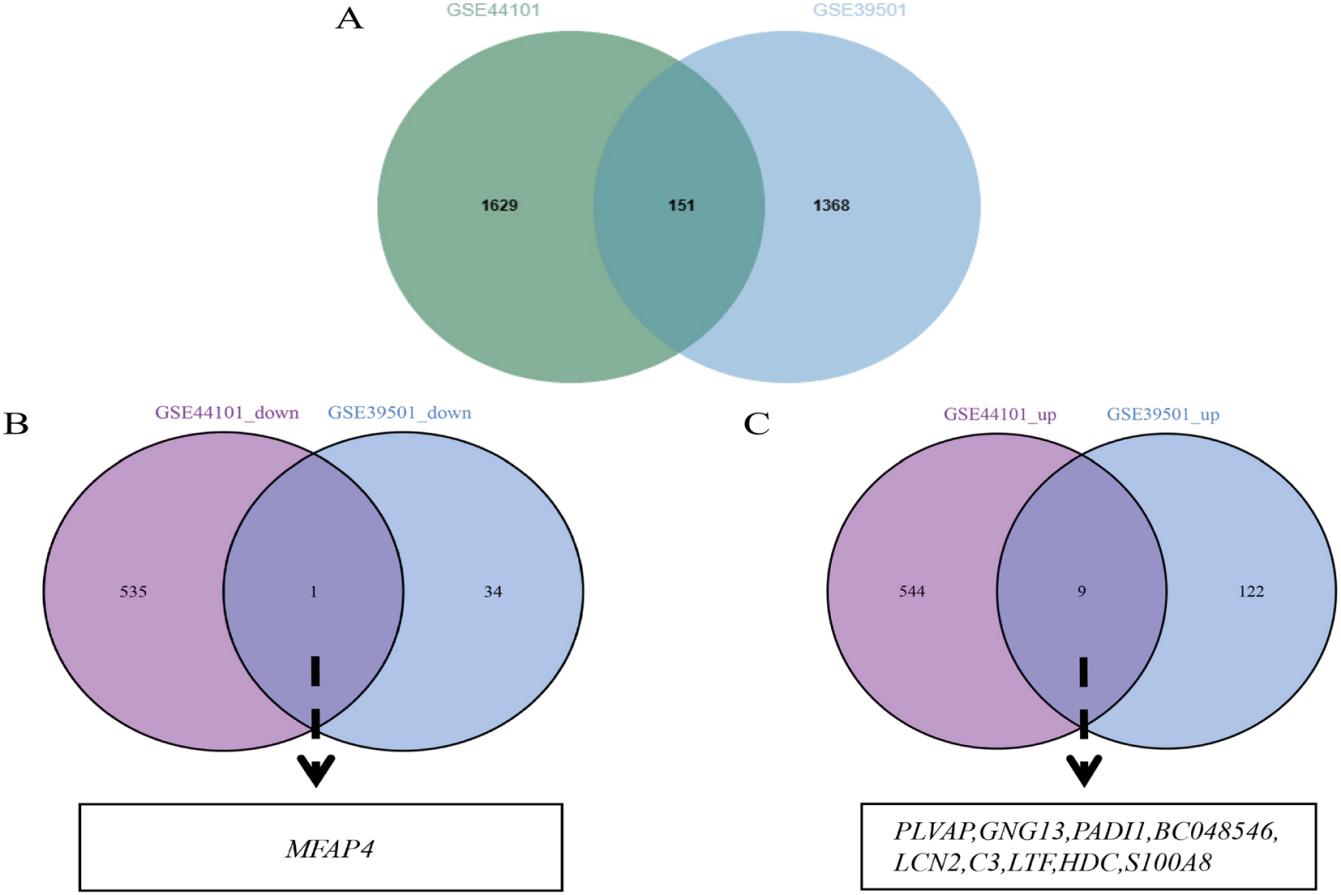
POF and DED co-morbidity-genes screening. (**A**) Intersecting WGCNA genes of POF and DED. Green color represents DED dataset and blue color represents POF dataset. (**B**) Intersecting down-regulated DEGs of POF and DED. Purple color represents DED dataset and blue color represents POF dataset. (**C**) Intersecting up-regulated DEGs of POF and DED. Purple color represents DED dataset and blue color represents POF dataset.

Differential gene expression analysis of GSE44101 and GSE39501 revealed 1089 DEGs in GSE44101, of which 553 genes were upregulated, and 536 genes were downregulated. A total of 166 DEGs were found in GSE39501, of which 131 were upregulated, and 35 were downregulated (Fig. 3). GSE44101 and GSE39501 had 10 common DEGs, of which nine genes were upregulated, namely BC048546, LCN2, LTF, PADI1, HDC, PLVAP, GNG13, S100A8, and C3, and one gene was downregulated, MFAP4, as shown in Fig. 4B, C.

### Enrichment analysis and functional annotation

**Figure 5:**
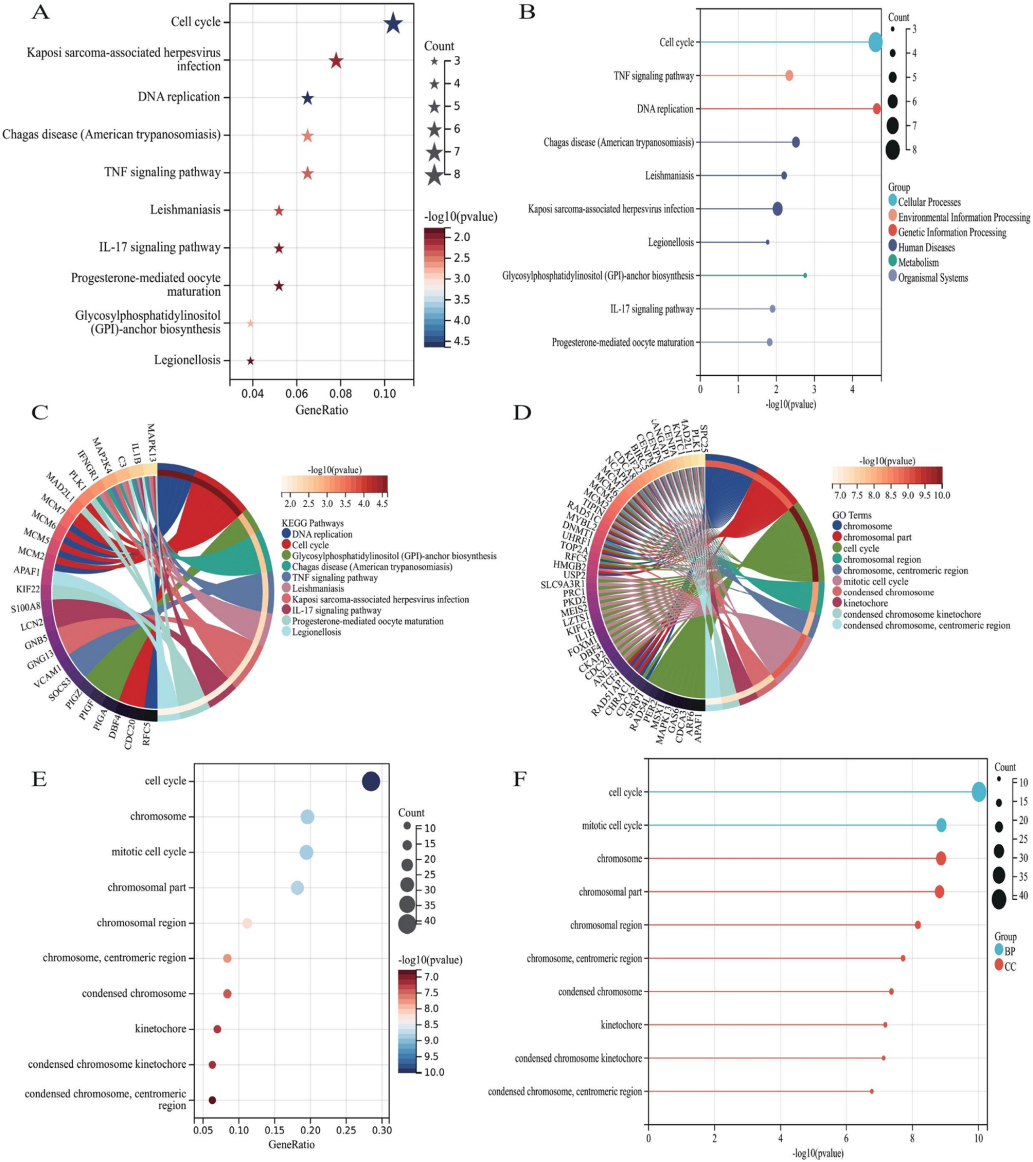
Enrichment analysis and Functional Annotation of comorbid genes between POF and DED. (**A**) Bubble diagram of KEGG enrichment analysis. (**C**) Histogram of KEGG enrichment analysis. (**D**) Circle diagram of KEGG enrichment analysis. (**E**) Bubble diagram of GO functional analysis. (**F**) Histogram of GO functional analysis. (**G**) Circle diagram of GO functional analysis.

After merging and removing duplicate genes, 158 DED and POF comorbid genes were obtained by combining all genes obtained from WGCNA and differential expression gene analysis. Genes were enriched using R to further clarify the functions of the screened genes and their roles in signaling pathways. The results showed that the identified genes were mainly enriched in DNA replication, cell cycle, glycosylphosphatidylinositol (GPI)-anchor biosynthesis, Chagas disease (American trypanosomiasis), TNF signaling pathway, leishmaniasis, Kaposi sarcoma-associated herpesvirus infection, IL-17 signaling pathway, progesterone-mediated oocyte maturation, and legionellosis, while its major genes functions were mainly in chromosome, chromosomal part, cell cycle, chromosomal region, chromosome, centromeric region, mitotic cell cycle, condensed chromosome, kinetochore, condensed chromosome kinetochore, condensed chromosome, centromeric region, as shown in Fig. 5.

### PPI network construction and modular analysis

**Figure 6:**
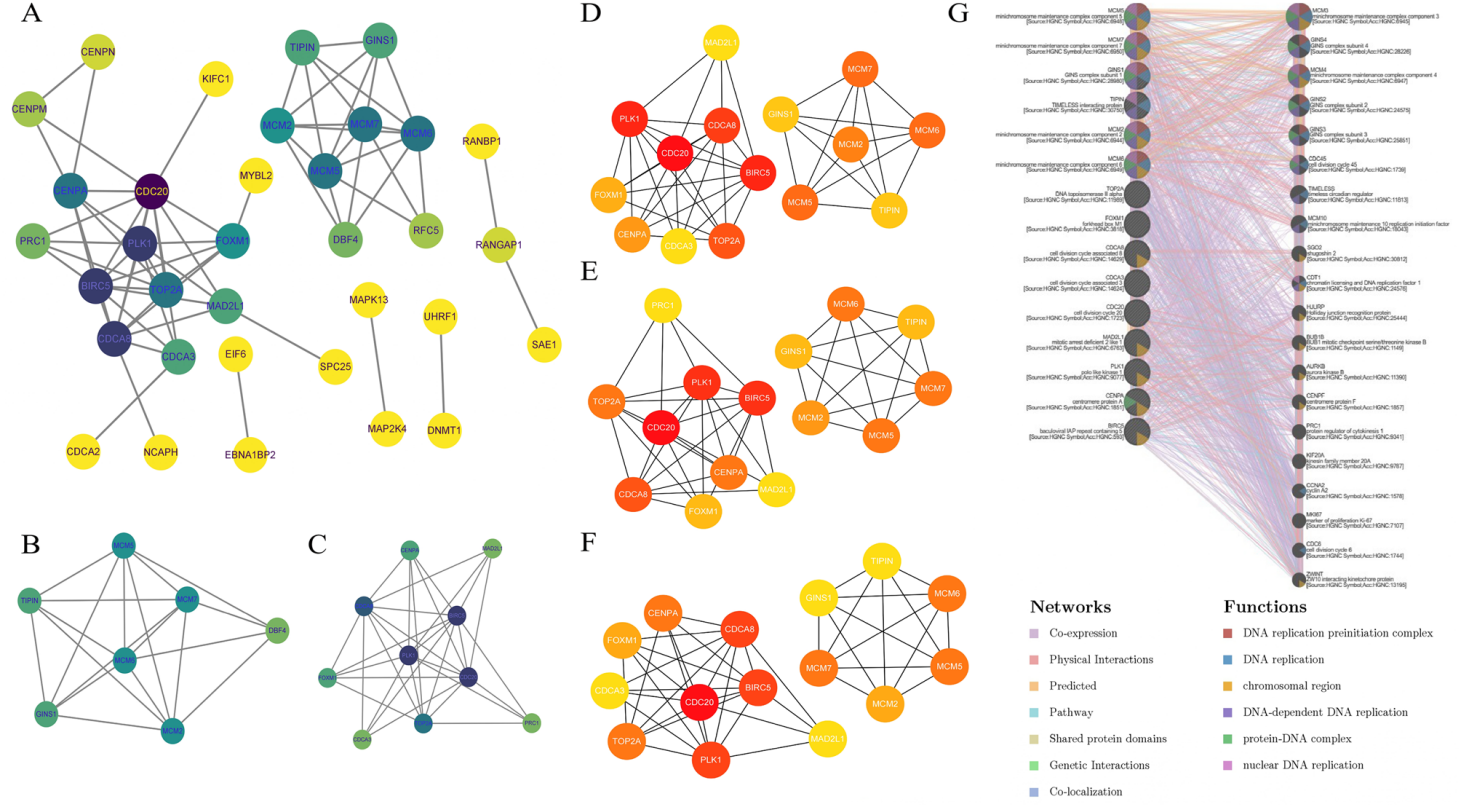
Identifcation and analysis of hub genes. (**A**) Protein–protein interaction network from String10.0. The purpler the node represents its higher significance, the yellower it is the opposite. (**B**) Cluster 1 from MCODE analysis of key genes. (**C**) Cluster 2 from MCODE analysis of key genes. (**D**) Identifcation of hub genes by CytoHubba of MCC ranking method. (**E**) Identifcation of hub genes by CytoHubba of MNC ranking method. (**F**) Identifcation of hub genes by CytoHubba of Dgree ranking method. (**G**) Hub genes and their co-expression genes were analyzed by GeneMANIA.

The genes obtained from WGCNA and differential expression genes analysis were imported into the STRING10.0 database, and the species was set as "homo sapiens" to obtain the genes comorbid in DED and POF. These genes were analyzed using PPI, and the key comorbid genes were screened, as shown in Fig. 6A. Two individual clustering modules of closely linked genes were extracted using the Molecular Complex Detection (MCODE) plugin (Fig. 6B, C). Cluster 1 contained 7 nodes and 19 edges. Cluster 2 contains 10 nodes and 32 edges.

### Selection and analysis of hub genes

Furthermore, network analysis using the CytoHubba plugin with MCC, MNC, and Degree value as reference standards identified 15 key hub genes, including CDC20, BIRC5, PLK1, CDCA8, TOP2A, MCM5, MCM6, MCM7, MCM2, CENPA, FOXM1, GINS1, TIPIN, MAD2L1, and CDCA3 (Fig. 6D, E, F). Subsequently, we evaluated the coexpression network and the associated roles of the hub genes using the GeneMANIA database. The network results identified 46.7% coexpression, 22.85% physical interactions, 18.15% predictions, 5.39% pathways, 2.51% shared protein domains, 2.27% genetic interactions, and 2.14% co-localization (Fig. 6G).

### Validation of hub gene expression

**Figure 7:**
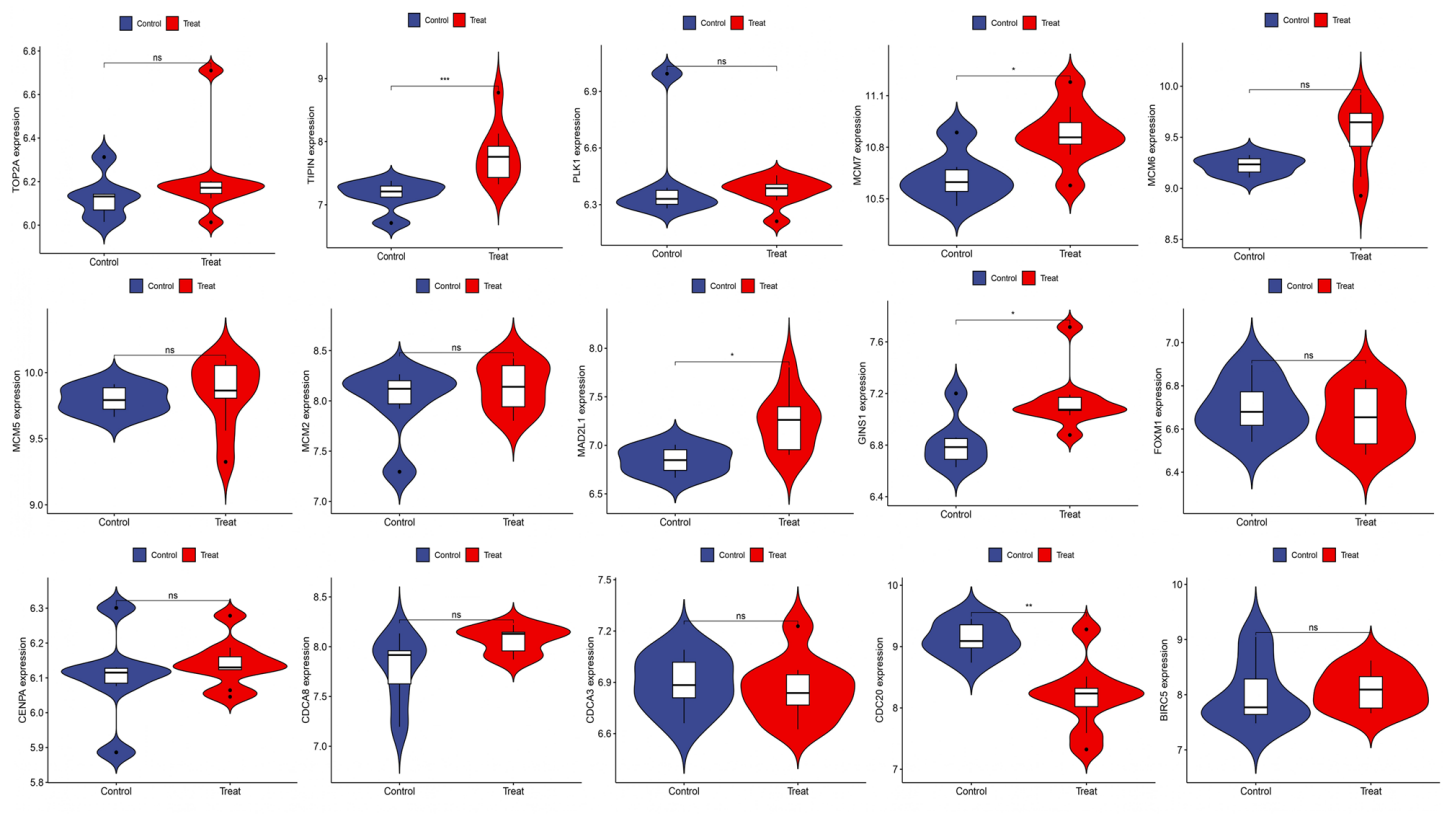
Validation of hub genes expression by POF dataset (GSE48873).

**Figure 8:**
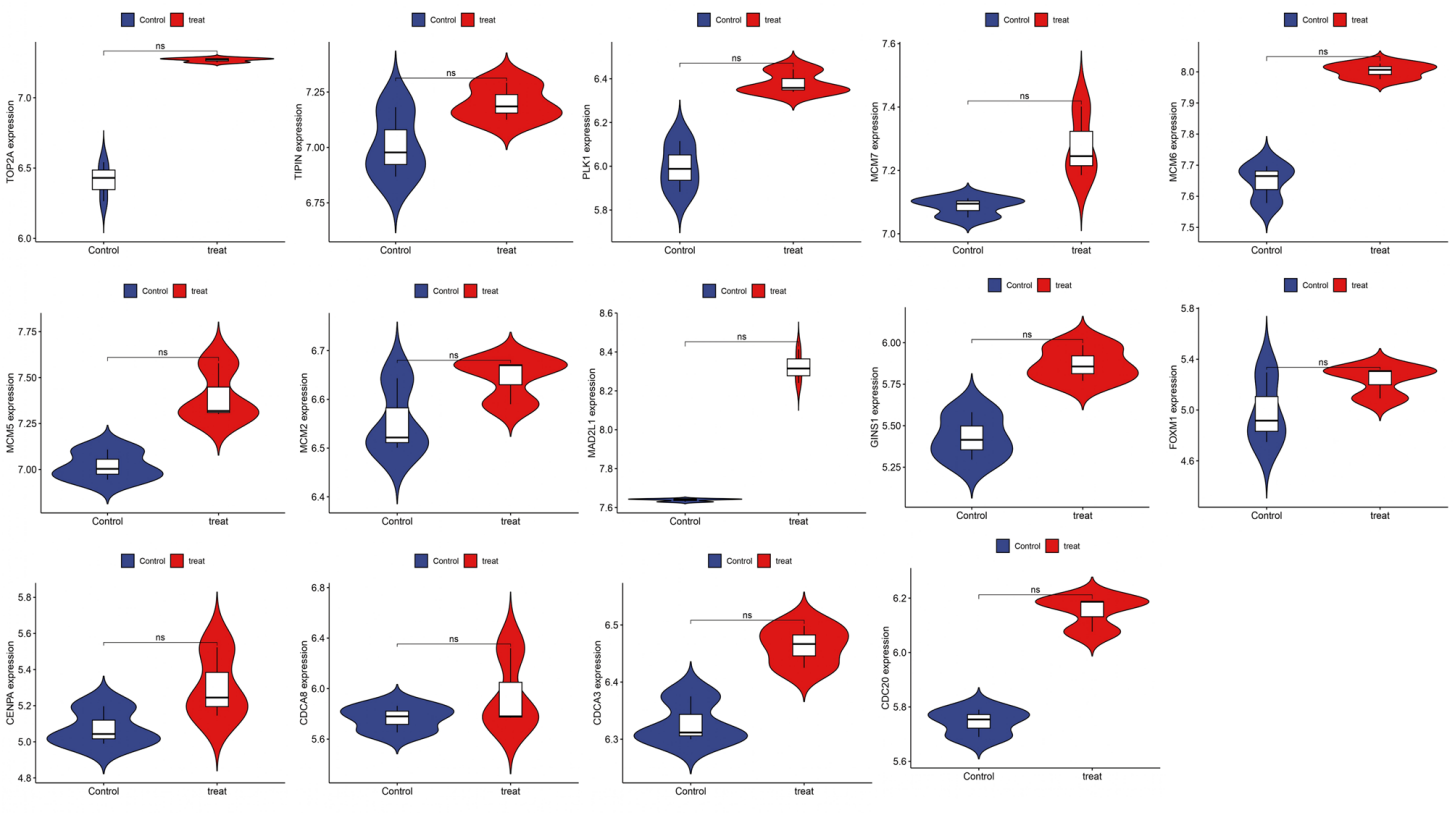
Validation of hub genes expression by DED dataset (GSE171043).

The expression levels of 15 key genes were validated using the POF-related dataset GSE48873 and the DED-related dataset GSE171043. The t-test was performed on each subset of the dataset. The significance level was set at *P* < 0.05. Herein, we found that PLK1, CDCA8, TOP2A, MCM5, MCM6, MCM2, CENPA, MCM7, TIPIN, GINS1, MAD2L1, CDCA3, BIRC5, FOXM1, and CDC20 were expressed in another POF-related dataset, in which MCM7, TIPIN, GINS1, and MAD2L1 expression levels were significantly higher (*P* < 0.05), and CDC20 expression levels were significantly lower (*P* < 0.05), as shown in Fig. 7. Additionally, CDC20, PLK1, TOP2A, MCM5, MCM6, MCM7, MCM2, CENPA, FOXM1, GINS1, TIPIN, MAD2L1, CDCA3, and CDCA8 were expressed in another DED-related dataset (Fig. 8). Therefore, we hypothesized that CDC20, CDCA8, PLK1, TOP2A, MCM5, MCM6, MCM7, MCM2, CENPA, FOXM1, GINS1, TIPIN, MAD2L1, and CDCA3 were comorbid in POF and DED. TIPIN, GINS1, MAD2L1, and CDC20 were also differentially expressed.

### Identification of common RNAs and construction of microRNAs (miRNAs)- long noncoding RNAs (lncRNAs) shared genes network

**Figure 9:**
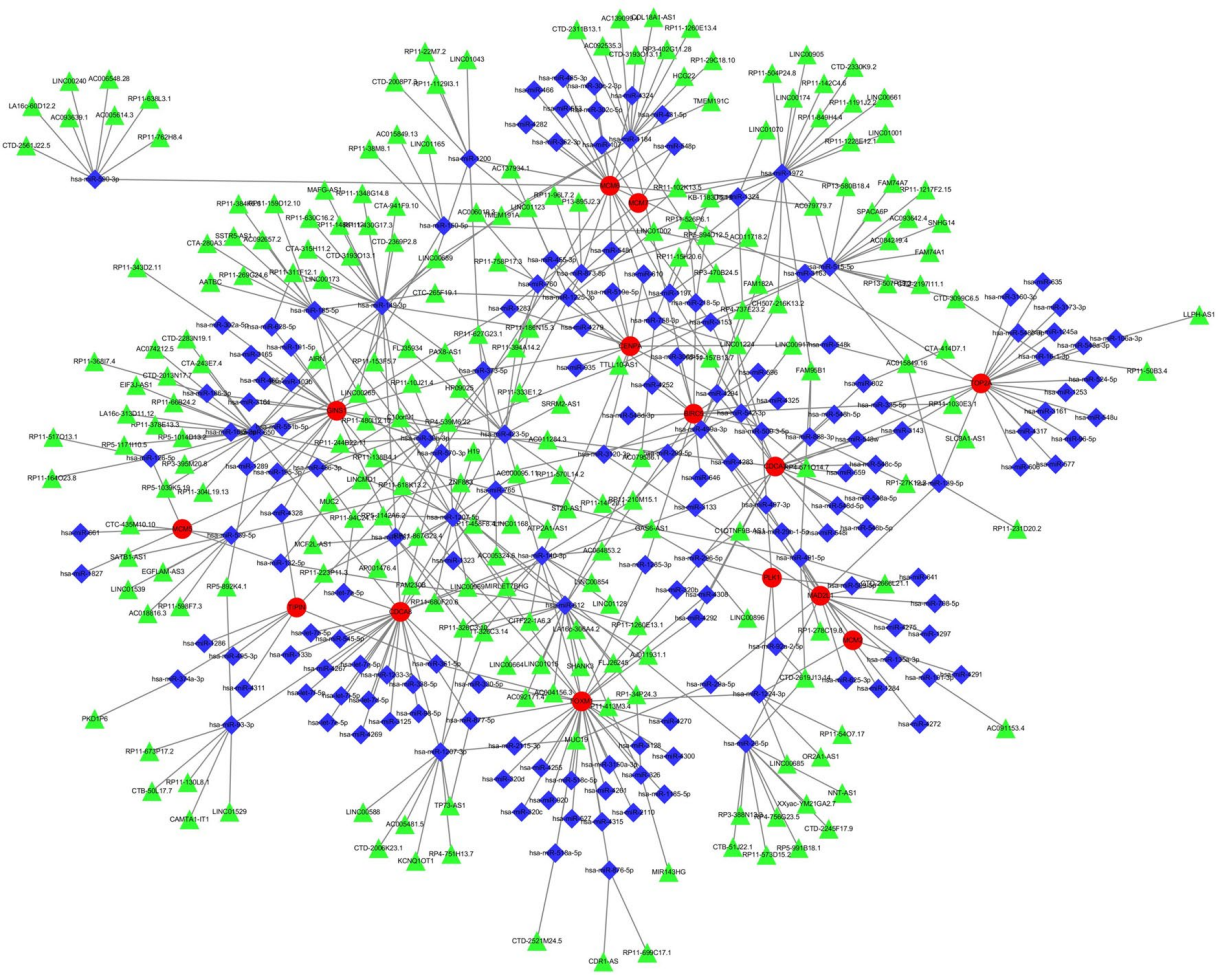
miRNAs-lncRNAs shared genes network. Red circles are mrnas, blue quadrangles are miRNAs, and green triangles are lncRNAs.

A total of 210 miRNAs and 296 lncRNAs associated with DED and POF were identified from the three databases, namely, miRanda, miRDB, and TargetScan. When the corresponding database matched the relevant miRNAs, the score was marked as 1. Upon matching all three databases were matched, a score of 3 points was assigned. miRNAs were matched using the SpongeScan database to obtain the corresponding lncRNA data. The miRNA-lncRNA-gene network was constructed by intersecting these non-coding RNAs with the shared genes. The network comprised 57 lncRNAs, 174 miRNAs, and some common genes, including the 14 hub genes (BIRC5, FOXM1, CDCA8, CDCA3, MCM6, CENPA, TOP2A, MAD2L1, PLK1, MCM5, TIPIN, MCM2, GINS1, and MCM7) (Fig. 9).

### Drug forecasting and drug-gene interaction analysis

**Figure 10:**
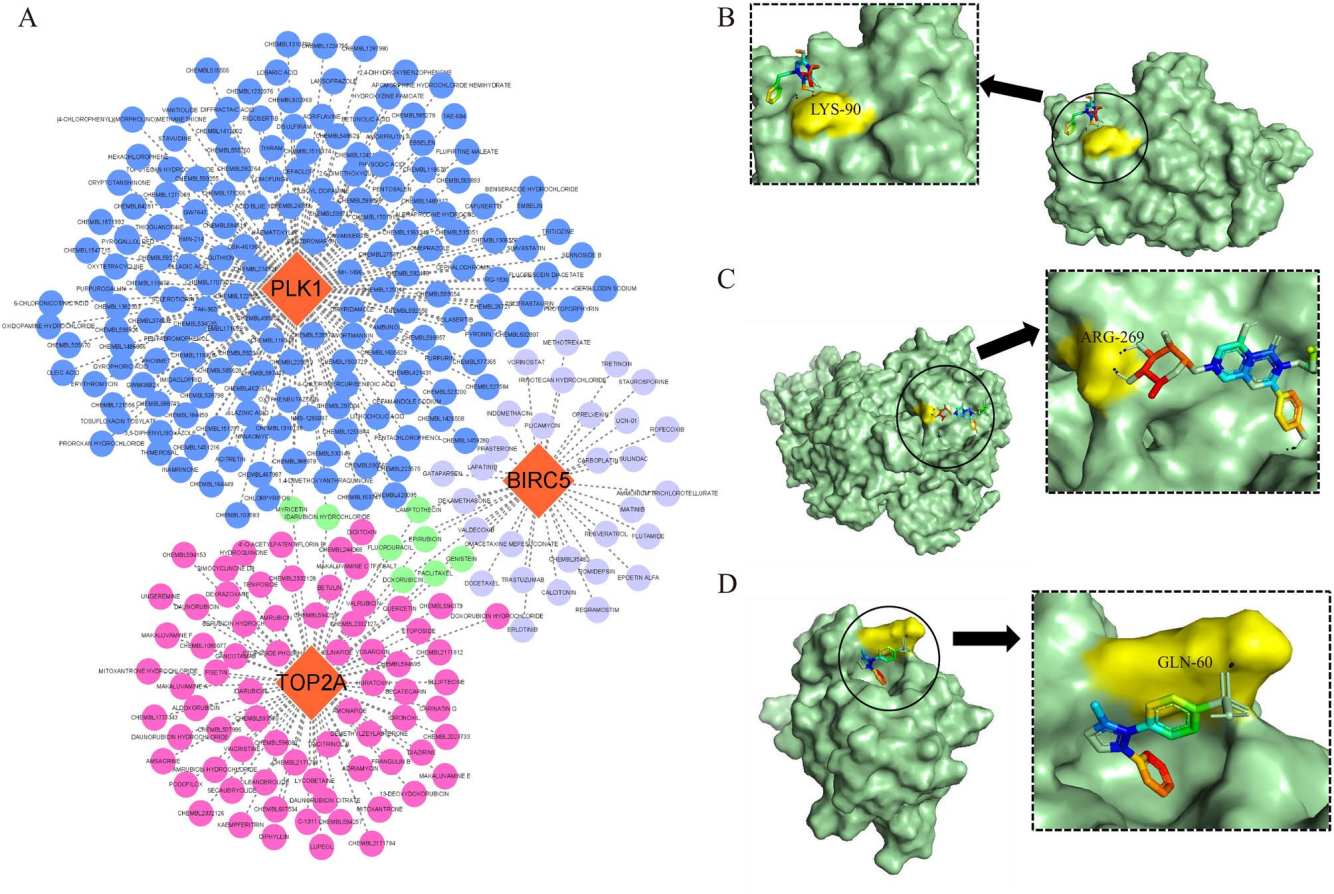
Drug-gene interaction network and docking analysis. (**A**) Drug-gene interaction network. Orange circles represent genes, blue circles represent predictive drugs for PLK1, purple circles represent predictive drugs for BIRC5, pink circles represent predictive drugs for TOP2A, and green circles represent predictive drugs associated with tow genes. (**B**) The docking of BIRC5 with VALDECOXIB, the docking energe is − 5.51 kJ/mol. (**C**) The docking of PLK1 with AMORFRUTIN A, the docking energe is − 1.19 kJ/mol. (**D**) The docking of TOP2A with KAEMPFERITRIN, the docking energe is − 1.84 kJ/mol.

The prediction was performed using the DGIdb database, and a total of 293 predicted drugs were obtained, of which BIRC5 corresponded to 36 predicted drugs, PLK1 corresponded to 176 predicted drugs, and TOP2A corresponded to 80 predicted drugs. We constructed a drug-gene interaction network (Fig. 10A) and found that idarubicin hydrochloride and myricetin were common candidates for TOP2A and PLK1. Doxorubicin, fluorouracil, paclitaxel, epirubicin, camptothecin, and genistein were common TOP2A and BIRC5 candidates. According to the above studies, we screened these drugs based on their interaction scores after excluding antineoplastic drugs. The drugs with the highest interaction scores for each hub gene are listed in Table 1. The BIRC5-predicted drug was valdecoxib, the PLK1-predicted drug was amorfrutin A, and the TOP2A-predicted drug was kaempferitrin. Molecular docking between the hub genes and their predicted drugs revealed that the binding energies of the hub genes and their counterparts were < 0, suggesting that both could bind spontaneously. It is generally believed that the lower the energy, the more stable the ligand-receptor binding conformation and the greater the possibility of action. Analysis of the molecular docking results showed that BIRC5 had the lowest binding energy with valdecoxib ( − 5.51 kJ/mol), indicating that the ligand and receptor have the most stable conformation. The docking results are shown in Fig. 10B, C, D.

**Table 1:** Drug-gene docking analysis.

Gene	Drugs	Interaction score	Molecular formula	3D Conformer	Molecular docking score (KJ/mol)
BIRC5	VALDECOXIB	1.72	C16H14N2O3S		− 5.51
PLK1	AMORFRUTIN A	0.09	C21H24O4		− 1.19
TOP2A	KAEMPFERITRIN	1.55	C27H30O14		− 1.84

### Machine learning model building

**Figure 11:**
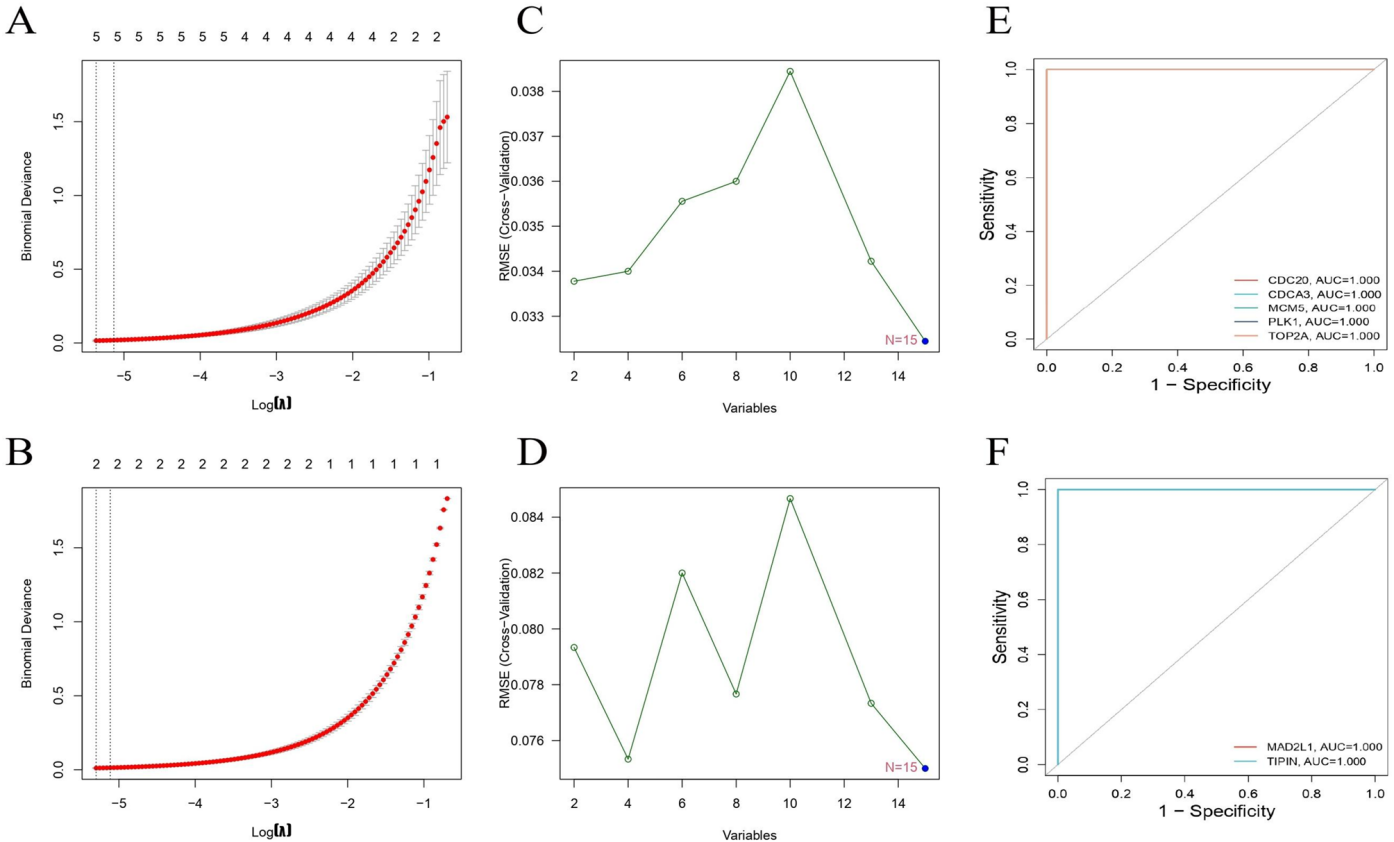
(**A**) Regression of the 15 shared genes using LASSO in GSE39501. (**B**) Regression of the 15 shared genes using SVM-RFE in GSE39501. (**C**) ROC of characteristic genes in GSE39501. (**D**) Regression of the 15 shared genes using LASSO in GSE44101. (**B**) Regression of the 15 shared genes using SVM-RFE in GSE44101. (**C**) ROC of characteristic genes in GSE44101.

The 15 identified genes were explored as potential markers using the least absolute shrinkage and selection operator (LASSO) regression algorithm and support vector machine recursive feature elimination (SVM-RFE) analysis (Fig. 11). In the LASSO regression algorithm analysis of hub genes in GSE39501, five featured genes, namely CDC20, CDCA3, MCM5, PLK1, and TOP2A, were considered as characteristic genes that significantly correlated with the samples. Likewise, in GSE44101, two featured genes, namely, MAD2L1 and TIPIN, were considered characteristic genes. Notably, in the SVM-RFE analysis, all hub genes in GSE39501 and GSE44101 were identified as characteristic genes. We combined these two results for receiver operating characteristic (ROC) analysis of hub genes, revealing that CDC20, CDCA3, MCM5, PLK1, TOP2A, MAD2L1, and TIPIN possessed good diagnostic performance as characteristic genes, with an area under the curve (AUC) of 1.

## Discussion

POF, also known as pathological ovarian aging, is a reproductive disorder that markedly affects women during their fertile years. The cardinal facets of ovarian aging include a diminishing count and compromised follicle quality, thereby resulting in menstrual cycle irregularities or outright amenorrhea^2^. This progressive decline in fertility ultimately culminates in a complete loss of reproductive capacity, concurrently entwined with the onset of multifaceted systemic ailments culminating in menopause. Women with POF have an increased susceptibility to diverse systemic and ocular disorders^13^. DED, a prevalent ocular affliction with global ramifications, is characterized by inadequate tear production and tears of suboptimal quality, precipitating discomfort, visual disturbances, and consequential harm to the ocular surface^16^. Various risk factors, such as age, hormonal fluctuations, and autoimmune conditions, have been identified in DED^10^. Some studies have suggested that DED is associated with comorbidities. Recent empirical data indicate an elevated prevalence of DED among individuals with POI when compared with those with normal ovarian function^12^. However, the underlying mechanisms and the potential interconnections between these two conditions remain unclear. Against the backdrop of an evolving narrative on tumorigenesis, the scientific focus is progressively veering toward non-oncological realms. The collective interplay between comorbid genes in POF and DED represents an uncharted territory awaiting further exploration. A nuanced examination of the distinctive patterns of comorbidity amid the progression of POF and DED may elicit crucial insights into their pathogenic roles, thereby fostering the development of precisely targeted therapeutic interventions.

In the current study, we conducted data mining and analyzed the comorbidity mechanism of POF and DED based on bioinformatics methods, such as WGCNA and DEGs. We used the CytoHubba plug-in to perform an in-depth analysis of the PPI network and identified 15 key hub genes: CDC20, BIRC5, PLK1, CDCA8, TOP2A, MCM5, MCM6, MCM7, MCM2, CENPA, FOXM1, GINS1, TIPIN, MAD2L1, and CDCA3. Accordingly, hub genes were mainly covered by the cell division cycle (CDC) and minichromosome maintenance (MCM) gene families. Several studies have found that CDC family genes are associated with apoptosis^17^, which induces cellular senescence owing to its apoptotic mechanism, with the induction of cell senescence possibly resulting in numerous ocular surface disorders, including DED^18^. Simultaneously, cellular senescence, as one of the key causes of DED, is associated with alterations in the structure and function of the lachrymal gland, such as atrophy and reduced protein secretion^19^, with a higher probability of lacrimal gland lesions in older females^20^. The MCM gene family is widely found in eukaryotes such as humans, plays a role in regulating cell division, and is a key factor in initiating DNA replication^21^. Abnormalities in DNA replication are key to inducing reduced tearing and episodes of DED, which is consistent with the results of gene enrichment analysis. Primordial follicle depletion or dysfunction is key to the development of POF caused by MCM5, increasing the proliferation of primary granulosa cells, which, in turn, promotes the activation of primordial follicles in neonatal mouse ovaries^22^. The growth and development of mouse oocytes were found to be associated with three proteins of the MCM family: MCM2, MCM6, and MCM7^23^. The expression levels of these pivotal genes were validated using the POF-related dataset GSE48873 and DED-related dataset GSE171043, suggesting that CDC20, CDCA8, PLK1, TOP2A, MCM5, MCM6, MCM7, MCM2, CENPA, FOXM1, GINS1, TIPIN, MAD2L1, and CDCA3 are potential comorbid genes in POF and DED. Notably, TIPIN, GINS1, MAD2L1, and CDC20 were differentially expressed. Meanwhile, to clarify the diagnostic performance of hub genes, we performed a rich machine-learning analysis, revealing that under the SVM-RFE algorithm, all 15 hub genes screened were characterized as genes. Using the LASSO algorithm and ROC analysis, CDC20, CDCA3, MCM5, PLK1, TOP2A, MAD2L1, and TIPIN showed good diagnostic performance. CDC20 is a key player involved in the cell cycle^24,25^. Silencing CDC20 expression was found to activate apoptosis^26^. In addition, CDC20 expression is closely related to oocyte development. Insufficient expression of CDC20 can suppress the development of oocytes^27^, and the abnormal development of oocytes inevitably leads to the occurrence of POF, which is consistent with the results of our analysis. Moreover, CDC20 can be regulated by estrogen and progesterone, which could offer a new strategy to improve the success rate of assisted reproduction^28^. Using bioinformatics analysis, CDC20 and TOP2A were found to be differentially expressed in POF, revealing low expression^29^. CDCA8 encodes Borealin/Desra B protein, which is an important component of the chromosome passenger complex^30^. The transcriptional activity of CDCA8 is increased in embryos, embryonic stem cells, and cancer cells, whereas it is weakly or unexpressed in normal tissues^31^. CDCA8 affects cell proliferation by regulating the cell cycle^32^. CDCA8 is also associated with cellular immune infiltration, and overexpression can lead to decreased cellular immune infiltration^33^. CDCA3, also known as mitotic entry trigger factor 1, has been identified in several studies and reportedly mediates cell-cycle progression^34^. TIPIN, a key factor in the cell cycle, is closely associated with DNA replication and interacts with the core circadian protein to form the Timeless-TIPIN complex, which is involved in normal DNA replication and maintains genomic stability^35^. During DNA replication, the Timeless-TIPIN complex moves along the replication fork through its checkpoint-adjustment or independent adjustment function to maintain the integrity and stability of the replication fork and facilitate the return of the DNA replication process to normal^36^. Downregulation of the timeless-TIPIN complex was shown to reduce the rate of DNA synthesis^37^. Disease manifestation is reflected by the fact that low TIPIN expression leads to apoptosis owing to aberrant DNA replication. Silencing of the highly expressed TIPIN inhibits cell proliferation. The timeless-TIPIN complex is closely related to the MCM family genes in the regulation of apoptosis^38^. MAD2L1 is a component of the mitotic spindle assembly checkpoint that prevents late-stage initiation until all chromosomes are correctly aligned at mid-stage. As a major mitotic checkpoint gene, MAD2L1 is closely associated with cellular replication; thus, high MAD2L1 expression can lead to abnormal cell proliferation. MAD2L1 knockdown reportedly accelerates the production of reactive active species (ROS), which mediates mitochondrial DNA damage^39^. Meanwhile, the inhibition of MAD2L1 disrupts mitochondria, and mitochondrial dysfunction leads to increased ROS production and inflammatory responses. Furthermore, mitochondrial dysfunction is a feature of cellular senescence, which also contributes to cellular senescence^40^. Therefore, it is crucial to alleviate abnormal apoptosis and senescence when treating comorbid POF and DED.

**Figure 12:**
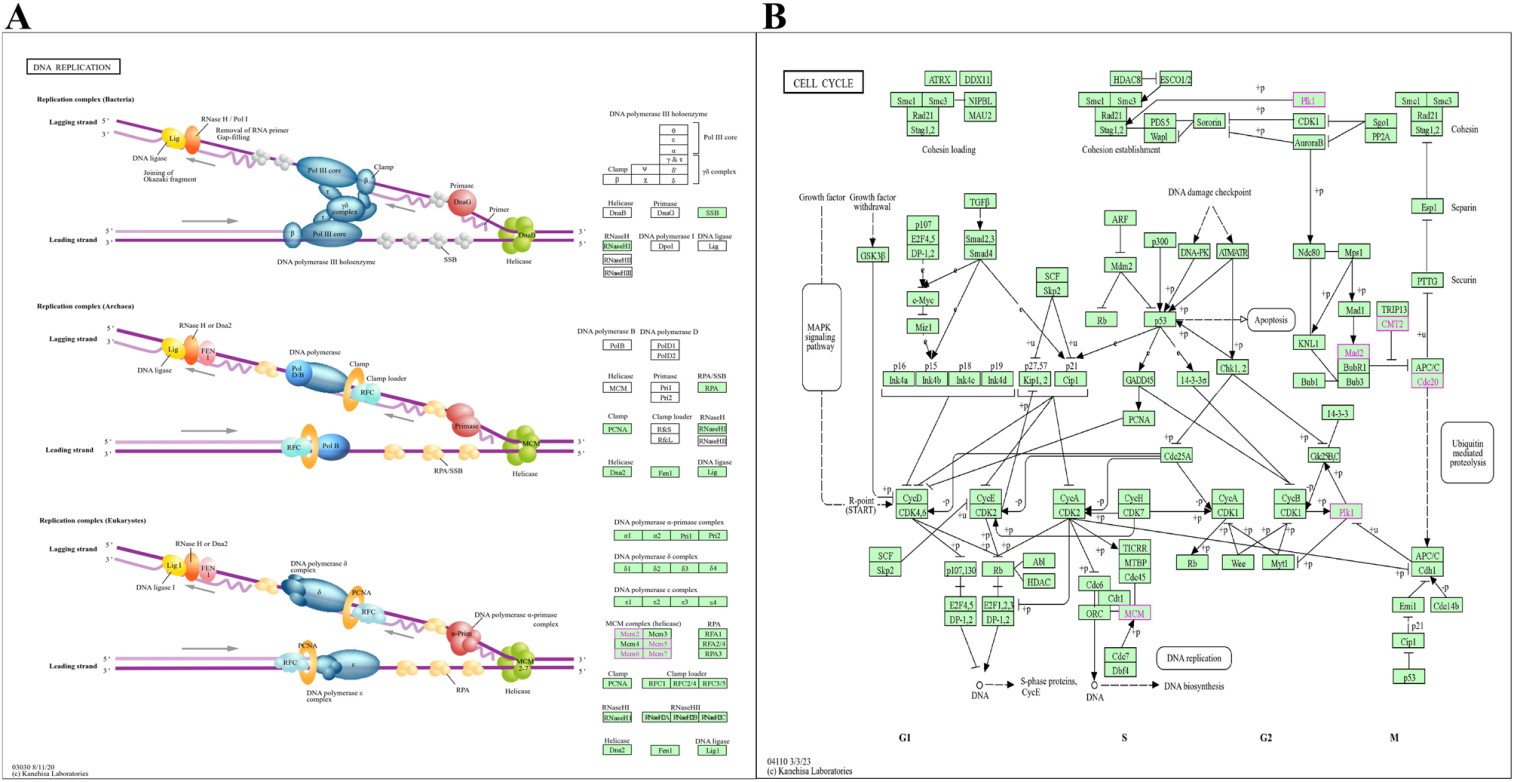
The key signaling pathways. (**A**) DNA replication. (**B**) Cell cycle.

After preliminary mining of the comorbid genes of POF and DED, we performed gene enrichment analysis and functional annotation and found that the comorbidity mechanism of POF and DED was predominantly related to DNA replication (Fig. 12A, a figure from the KEGG pathway database^41^), cell cycle (Fig. 12B, a figure from the KEGG pathway database^41^), and the IL-17 signaling pathway. Researche shows that cisplatin reduces DNA replication and thus alleviates tumorigenesis; however, it also affects ovarian function, leading to POF^42^. Additionally, abnormalities in DNA replication can lead to the development of DED. The proliferation of vesicular cells due to abnormalities in DNA replication and the cell cycle can reduce tearing and episodes of DED^43^. The IL-17 family of proteins plays crucial roles in the development of inflammatory diseases. IL-17 can drive autoimmune diseases^44^. IL-17 was found to be closely related to the development of DED^45^, as well as an effector of immune cells Th-17^46^, given that Th17 is crucially involved in autoimmune diseases and the body's defense response, and IL-17 in the body is typically involved in inflammatory reactions and autoimmune diseases. This has important research significance for autoimmunity-related POF. Despite the promising potential of these mechanisms, it is crucial to recognize the current gaps in our understanding of their regulatory mechanisms within the comorbid framework of DED and POF. Further investigations are necessary to elucidate their precise roles and interactions with transcription factors that are pivotal for the comorbid regulation of DED and POF. These mechanistic insights will considerably advance our comprehension of the biological processes underlying the development and progression of comorbid DED and POF, thereby opening new avenues for targeted therapeutic interventions.

By leveraging the identified hub genes, we constructed a comprehensive network of shared RNAs encompassing both miRNAs and lncRNAs. This intricate network comprised 57 lncRNAs, including CTA-414D7.1, LINC01043, GAS6-AS1, RP11-326C3.10, RP11-10J21.4, and MUC19. Additionally, it incorporated 174 miRNAs, including hsa-miR-802, hsa-miR-1200, hsa-miR-765, hsa-miR-612, hsa-miR-767-5p, hsa-miR-570-3p, hsa-miR-1207-3p, and hsa-miR-326, hsa-miR-218-5p. The construction of this shared gene network represents a pivotal step in comprehensively clarifying the molecular interactions and regulatory mechanisms underlying complex diseases. Integrating lncRNAs and miRNAs into a unified network framework enables the exploration of their synergistic roles and potential regulatory crosstalk in disease pathology. This network serves as a valuable resource for future research, providing a foundation for investigating the functional relevance of these noncoding RNAs in the context of comorbid POF and DED. Although direct evidence linking these RNAs with POF or DED is lacking, several studies have identified a regulatory role for their expression in ovarian tissues. The main mechanism may involve the regulation of cell proliferation and apoptosis. For example, microRNA-802 was found to suppress the growth of epithelial ovarian cancer cells by regulating cell proliferation and apoptosis in the ovarian tissue^47^. Additionally, has-miR-218-5p was highly expressed in HPV-infected ovarian tumor tissues^48^ lUCAT1 promotes the expression of HOXA13 by inhibiting hsa-miR-612, which ultimately leads to ovarian cancerdevelopment^49^. Silencing of BRD4 by hsa-miR-765 resulted in substantial anti-ovarian cancer activity^50^. Future research should focus on elucidating the specific functions and interactions between lncRNAs and miRNAs within the network. Detailed functional analyses and experimental validations are imperative to uncover their roles in gene regulation, signaling pathways, and disease progression. By advancing our understanding of these shared RNAs, we can identify novel biomarkers for early diagnosis and develop targeted therapeutic strategies to mitigate the effects of POF and DED. This integrative approach sheds light on the complex regulatory networks that drive disease comorbidity and paves the way for innovative interventions in precision medicine.

Further analysis revealed that the potential therapeutic agents used were valdecoxib for BIRC5, amorfrutin A for PLK1, and kaempferitrin for TOP2A. Valdecoxib is a non-steroidal anti-inflammatory drug that reportedly inhibits phagocytosis by macrophages^51^ and can prevent apoptosis induced by endoplasmic reticulum stress^52^. BIRC5 is a cell cycle-related regulator of apoptosis^53^. Valdecoxib has high structural stability with BIRC5 docking, as well as a high potential to act via a mechanism possibly related to anti-apoptosis. Amorfrutin A exerts potent anti-inflammatory effects and can be used to treat inflammatory diseases^54^. PLK1 is closely associated with inflammation and plays a role in inflammatory response via ROS-mediated NLRP3 inflammatory vesicles. Kaempferitrin is the main compound in Chenopodium ambrosioides extract, a natural herbal ingredient with anti-inflammatory and antioxidant activities^55^. TOP2A, topoisomerase II α, was found to be closely associated with sepsis-induced acute lung injury, which involves a variety of responses, including inflammatory responses and apoptosis^56^. Importantly, the mechanism underlying comorbid POF and DED cannot be distinguished from the occurrence of an inflammatory response, especially when an autoimmune-related inflammatory response is common. Moreover, amorfrutin A, PLK1, kaempferitrin, or TOP2A can be explored to achieve anti-inflammatory responses.

There have been noteworthy efforts to utilize bioinformatics analyses to elucidate the links between metabolic processes and eye and gynecological diseases. Wang et al. highlighted MMP1 and MMP9 as promising predictive indicators and therapeutic targets for uveal melanoma^57^. Hu et al. developed a bioinformatics model for thyroid-eye disease and identified 11 hub genes (ATP6V1A, PTGES3, and PSMD12) with potential implications in these conditions^58^. In a seminal investigation^59^. Qin et al. reported a breakthrough with the identification of a CXCL10-based model exhibiting excellent diagnostic precision for POF. This model, encapsulating CXCL10 alongside Itga2 and Raf1, not only demonstrated robust diagnostic accuracy but also stands poised as a prospective and invaluable diagnostic biomarker. Thus, the collective expression profiles of these genes represent a reservoir of pertinent diagnostic information for POF. Furthermore, comprehensive research endeavors by Liu et al. shed light on a distinct set of six pivotal genes: SRSF1, PDIA5, NEURL1B, UNK, CELF2, and CFL2. Augmenting this discovery, the investigation also identified five hub miRNAs—hsa-miR-27a-3p, hsa-miR-24-3p, hsa-miR-22-3p, hsa-miR-129-5p, and hsa-miR-17-5p. Intriguingly, these findings suggest a regulatory nexus wherein the expression of these key genes appears to be orchestrated by two central hub miRNAs: hsa-miR-27a-3p and hsa-miR-17-5p^60^. These findings not only augment our understanding of the intricate molecular landscape associated with POF but also herald a paradigm shift in diagnostic possibilities. However, the exploration of the mechanisms of co-morbidity between the two diseases, POF and DED, is still in a lacklustre stage.

The objective of the current study was to bridge this gap using a novel methodology. We enriched our analysis by incorporating extensive POF and DED datasets from a continuously refined GEO database. Our predictive model for comorbidity provides a theoretical framework and charts a course for forthcoming metabolic research and the therapeutic modulation of metabolic perturbations in POF and DED. The novelty of comorbidity research lies in the fact that it breaks away from the traditional concept of treating different diseases as independent entities in the field of medicine, and takes into account the correlation and interaction between different diseases. By studying the phenomenon of comorbidity, the interactions and influences between diseases can be better understood, providing more comprehensive guidance for diagnosis and treatment in clinical medicine. By taking multiple factors into account, the complexity and diversity of diseases can be better grasped, providing more effective strategies for prevention and treatment. Besides, the novelty of this study is mainly reflected in the use of cutting-edge and rich bioinformatics analyses in order to comprehensively and systematically reveal the association and interactions between POF and DED. Through big data analysis, potential associations hidden in massive data were mined to provide more precise and in-depth analyses for co-morbidity studies. Meanwhile, through the mining of mechanisms combined with bidirectional causality, it provides theoretical basis and practical guidance for in-depth understanding and intervention of POF and DED comorbidity. Nevertheless, our investigation has some limitations. The fundamental mechanisms underlying POF and DED require further empirical validation through comprehensive in vitro and in vivo studies. In addition, the prognostic implications of comorbid genes remain elusive, presenting a valuable opportunity for future research. However, the intricate relationship between these comorbid genes in POF and DED remains elusive. Unraveling this relationship could provide critical insights into the pathophysiology of these conditions and reveal novel targets for therapeutic intervention. This represents fertile ground for future inquiry, with the potential to substantially advance our understanding of the molecular underpinnings and prognostic markers of comorbid POF and DED. Future investigations should focus on detailed functional analyses of the identified hub genes and their interactions. This includes exploring their roles in key signaling pathways, regulatory networks, and disease processes. Additionally, large-scale clinical studies are necessary to validate the prognostic value of these genes and translate these findings into clinical practice.

## Materials and methods

### Data source and preprocessing

GEO (http://www.ncbi.nlm.nih.gov/geo/) is an extensive and publicly available gene expression database created and maintained by the National Center for Biotechnology Information (NCBI), containing high-throughput gene expression data submitted by research institutions worldwide. We used the inputs “dry eye” and “premature ovarian failure” to retrieve gene expression datasets for DED and POF. Inclusion criteria were as follows: (i) the sample size of each group was ≥ 3, (ii) the samples in the normal and control groups were included, and (iii) the two datasets used for analyses were of the same species, and the dataset used for validation and analyses, respectively, should preferably be of different species. (iv) The data set must have access to raw and complete data that can be exported for analysis. After screening the dataset, we preprocessed the matrix files obtained from the platform and matched the probes with their gene symbols according to the corresponding annotation files of the platform. Finally, we obtained gene matrices with row names as gene symbols and column names as sample names for subsequent analyses.

### WGCNA

WGCNA considers biological functions as a whole using a gene network construction algorithm based on the expression similarity between genes. To identify DED- and POF-related gene coexpression modules, we used gene expression profiles to eliminate the top 50% genes with the smallest MAD and removed outlier genes and samples using the good-samples-genes method of the R package WGCNA, which was used to construct scale-free coexpression networks using a power function to construct a weighted neighbor-joining matrix. β is a soft-threshold parameter that emphasizes inter-gene strong correlations and penalizes weak correlations. Genes with similar expression profiles were grouped into gene modules, and the minimum size of the gene dendrogram was 30. The sensitivity was set to 3. To further analyze the modules, we computed the similarity of genes characterized by the modules, selected the cut lines of the module dendrograms, and merged the modules with distances < 0.25. Modules with high correlation coefficients for DED and POF were selected, and genes from these modules were obtained for further analysis. We used an online tool (https://jvenn.toulouse.sra.fr/app/example.html) to determine the intersection of obtained genes using a Venn diagram, which can be used to obtain genes coexpressed in DED and POF.

### Identification of DEGs

DEGs in DED and POF were identified using the R software limma package. Specifically, datasets were processed, converted into expression matrices, and grouped. The datasets were normalized and analyzed by using the limma software package and set adjusted with *P* < 0.05 and log2|FC (fold change)|≥ 1. The analysis identified DEGs, along with relevant information regarding their upregulated and downregulated expression. Considering the intersection between upregulated and downregulated expressed genes using the Venn diagram, genes common to DED and POF, respectively, were identified.

### Enrichment analysis and functional annotation

To further elucidate the functions of the screened genes, we performed KEGG enrichment analysis and GO functional annotation of genes associated with the comorbidity of DED and POF, which were obtained using WGCNA and DEG analyses. For gene set functional enrichment analysis, we employed the KEGG rest API (https://www.kegg.jp/kegg/rest/keggapi.html) to obtain the latest gene annotations of the KEGG Pathway, and the R package clusterProfiler (version 3.14.3) to perform enrichment analysis. Enrichment analysis was performed with GO annotations of genes in the R package org.Hs.eg.db (version 3.1.0), including biological processes (BP), cellular components (CC), and molecular functions (MF), to obtain gene set enrichment results. A minimum gene set of 5 and a maximum gene set of 5000 were set, with *P* < 0.05 and *FDR* < 0.1 deemed statistically significant.

### PPI network construction and modular analysis

To clarify the interaction relationship between the genes derived from the WGCNA and DEG analyses, STRING (https://string-db.org), an online tool for retrieving interacting genes, was used to construct a PPI network. The species was set as "homo sapiens,” and the minimum required interaction score was set as high confidence (0.9), with obtained protein data imported into Cytoscape (http:// www.cytoscape.org; version 3.8.2) for PPI network analysis and visualization. Network analysis was then performed using relevant tools (Analyze Network). The PPI network was analyzed for key functional modules using the MCODE plug-in to obtain closely functioning subnetworks in the network, with the following specific parameters: degree cutoff = 2, max depth = 100, node score cutoff = 0.2, and K-core = 2.

### Identification and analysis of hub genes

The PPI genes were analyzed according to the degree-value algorithm using the CytoHubba plugin to understand the importance of individual genes in this network and identify hub genes. Using MCC, MNC, and Degree values as reference standards, we selected the top 15 hub genes and used GeneMANIA (https://genemania.org/) to generate the coexpression networks for identified hub genes.

### Validation of hub genes

To further strengthen the confidence of the results, we used R software to validate the expression of hub genes using the GSE171043 and GSE48873 datasets. Statistical significance was set at *P* < 0.05.

### Identification of common miRNAs and lncRNAs

Noncoding RNA transcripts, such as miRNAs and lncRNAs, play crucial roles in genetic regulation^61^. MiRNAs influence gene expression by either enhancing or inhibiting mRNA degradation and translation^62^. Conversely, lncRNAs are noncoding RNA molecules that typically comprise ~ 200 nucleotides and regulate various physiological and biochemical cellular processes by mediating chromosomal changes, transcriptional activation, and interference^63^. Recent studies have highlighted the extensive crosstalk between miRNAs and lncRNAs, which involves competition for binding between miRNAs, lncRNAs, and other regulatory targets^64^. Notably, competitive endogenous RNAs have been identified in which lncRNAs function by sequestering miRNAs. Therefore, in this study, we aimed to investigate whether specific miRNAs and lncRNAs exhibit shared regulatory mechanisms and developmental processes in DED and POF using the Perl software.

### Drug forecasting and drug-gene interaction analysis

To further understand the value of hub genes in clinical settings, we utilized the DGIdb database (https://dgidb.genome.wustl.edu/) for drug prediction and their interactions with the identified hub genes, thus exploring the treatment of comorbid POF and DED. Docking was performed after converting the compound and target protein formats to *pdbqt format using AutoDock software, setting up the folder and working directory, respectively, placing the prepared files, ensuring that the software was running, and adopting the default parameters of AutoDock Vina for docking. After obtaining the docking results, the docking results were converted from *pdbqt format to *pdb format using Open Babel (https://openbabel.org/) and then imported into PyMOL (https://pymol.org/2/) for visualization.

### Machine learning model building

To further explore the diagnostic value of intersecting genes, we constructed a reliability predictive model using machine learning. Regression analysis was performed using LASSO with the glmnet package in R. Hub gene expression values in the GSE44101 and GSE39501 datasets were used as predictor variables, and their sample groups were used as response variables. Cross-validation (cv) of the model was performed using the cv.glmnet function, and CV plots were plotted to determine the optimal regularization parameters. Based on the coefficients of the LASSO regression, genes with non-zero coefficients were identified as selected signature genes. Next, we employed the sophisticated SVM-RFE algorithm with the e1071 package to construct another machine-learning model. These analyses provided a more nuanced understanding of these genes. Subsequently, we performed ROC analysis to construct models for assessing the predictive performance of the results and the diagnostic value of a factor. AUC values ˃ 0.5 and approaching 1 indicate high diagnostic accuracy. AUC values between 0.5 and 0.7 indicate low accuracy, 0.7 to 0.9 moderate accuracy, and ˃ 0.9 high accuracy. We used the R package pROC (v1.17.0.1) to perform the ROC analysis and obtain AUC values. Specifically, we obtained the disease status of the patient along with gene expression and evaluated the AUC and confidence intervals to clarify the diagnostic value of the genes in the disease.

## Conclusion

The pathobiology of DED and POF involves an intricate tableau of multifactorial interactions encompassing a wide array of gene targets, pathways, signaling modalities, and regulatory frameworks. These components engage in a complex, often bidirectional interplay that exhibits both synergistic and antagonistic effects. Comorbid genes, including CDC20, BIRC5, PLK1, CDCA8, TOP2A, MCM5, MCM6, MCM7, MCM2, CENPA, FOXM1, GINS1, TIPIN, MAD2L1, and CDCA3, with processes encompassing DNA replication, the cell cycle, and the IL-17 signaling pathway, are central to this biological mosaic and are pivotal in catalyzing the biosynthesis of a spectrum of critical entities. The spotlight in this constellation of genes falls on CDC20, MAD2L1, and TIPIN, which is closely related to abnormal apoptosis and senescence. The functionality of these genes, whether in the activation or inhibition of metabolic pathways, is emblematic of the metabolic versatility integral to the pathogenesis of DED and POF.

## Data Availability

The datasets presented in this study can be found in GEO (https://www.ncbi.nlm.nih.gov/geo/). GSE44101, GSE39501, GSE171043, GSE48873 datasets were downloaded from the GEO database.

## Abbreviations

POF: Premature ovarian failure
POI: Premature ovarian insufficiency
DED: Dry eye disease
DES: Dry eye syndrome
OSD: Ocular surface disease
DTS: Dysfunctional tear syndrome
GEO: Gene Expression Omnibus
WGCNA: Weighted gene co-expression network analysis
DEGs: Differentially expressed genes
KEGG: Encyclopedia of Genes and Genomes
GO: Gene ontology
BP: Biological process
CC: Cellular component
MF: Molecular function
PPI: Protein–Protein interation
MCODE: Molecular complex detection
CDC: Cell division cycle
MCM: Minichromosome maintenance
MCC: Maximal clique centrality
MNC: Maximal neighborhood component
LASSO: Least absolute shrinkage and selection operator
SVM-RFE: Support vector machine recursive feature elimination
CV: Cross-validation
ROC: Receiver operating characteristic
AUC: An area under the curve
